# Transcriptome Analysis of Proximal Tubular Cells (HK-2) Exposed to Urines of Type 1 Diabetes Patients at Risk of Early Progressive Renal Function Decline

**DOI:** 10.1371/journal.pone.0057751

**Published:** 2013-03-07

**Authors:** Krzysztof Wanic, Bozena Krolewski, Wenjun Ju, Grzegorz Placha, Monika A. Niewczas, William Walker, James H. Warram, Matthias Kretzler, Andrzej S. Krolewski

**Affiliations:** 1 Research Division, Joslin Diabetes Center, Harvard Medical School, Boston, Massachusetts, United States of America; 2 Department of Medicine, Harvard Medical School, Boston, Massachusetts, United States of America; 3 Department of Metabolic Diseases, Jagiellonian University, Krakow, Poland; 4 Division of Nephrology, Department of Internal Medicine, University of Michigan, Ann Arbor, Michigan, United States of America; 5 Department of Hypertension, Warsaw Medical University, Warsaw, Poland; Children's Hospital Boston/Harvard Medical School, United States of America

## Abstract

**Background:**

In patients with Type 1 Diabetes (T1D) who develop microalbuminuria, progressive decline in glomerular filtration rate (GFR) may be initiated by leakage into the urine of toxic proteins (txUPs). This study tested this hypothesis.

**Methods:**

After archiving baseline urine, we followed T1D patients with microalbuminuria for 8–12 years to distinguish those in whom GFR declined (Decliners) and those in whom it remained stable (Non-decliners). Human proximal tubular cells (HK-2 cells) were grown in serum-free medium enriched with pooled urines from Decliners or Non-decliners. We determined genome-wide expression profiles in extracted mRNA.

**Results:**

The two pooled urines induced differential expression of 312 genes. In terms of gene ontology, *molecular functions* of the 119 up-regulated genes were enriched for protein binding and peptidase inhibitor activities. Their *biologic processes* were enriched for defense response, responses to other organisms, regulation of cellular processes, or response to stress or stimulus, and programmed cell death. The 195 down-regulated genes were disproportionately represented in *molecular functions* of cation binding, hydrolase activity, and DNA binding. They were disproportionately represented in *biological processes* for regulation of metabolic processes, nucleic acid metabolic processes, cellular response to stress and macromolecule biosynthesis. The set of up-regulated genes in HK-2 cells overlaps significantly with sets of over-expressed genes in tubular and interstitial compartments of kidney biopsies from patients with advanced DN (33 genes in one study and 25 in the other compared with 10.3 expected by chance, p<10^−9^ and p<10^−4^, respectively). The overlap included genes encoding chemokines and cytokines. Overlap of down-regulated genes was no more than expected by chance.

**Conclusions:**

Molecular processes in tubules and interstitium seen in advanced diabetic nephropathy can be induced *in vitro* by exposure to urine from patients with minimal microalbuminuria who subsequently developed progressive renal function decline, presumably due to putative txUPs.

## Introduction

Moderate elevation of urinary albumin excretion, referred to as microalbuminuria (MA), is the earliest indicator of diabetic nephropathy (DN) in Type 1 diabetes (T1D) [1]. However, while MA is a very sensitive marker, it is not specific for the disease process that leads to renal failure. In two Joslin studies of the natural history of MA, only a third of the patients experienced renal function loss as reflected in a progressive decline in the glomerular filtration rate (GFR) during the subsequent 4–12 years of follow-up [2]–[4]. Renal function was stable and normal in the rest. We designate this decline as early progressive GFR loss because it began soon after onset of MA when GFR was normal or even elevated. Nevertheless, it persisted during follow-up and eventually led to impaired renal function and end-stage renal disease (ESRD) [2], [3].

Investigation of systemic factors has identified several that contribute to the risk of early GFR loss, such as older age, elevated HbA1c, elevated blood pressure, high normal values of serum uric acid and elevated levels of circulating TNF-Rs [2]–[4]. In addition to systemic factors, we have been searching for urinary markers associated with early GFR loss. For example, as reported by Wolkow et al., urinary concentrations of IL-6, IL-8, MCP-1, IP-10 and MIF are elevated in those patients with MA who later developed early GFR loss (Decliners) in comparison with those whose renal function remained stable and normal (Non-decliners). Importantly, when the urine samples were taken, concentrations of these chemokines were similar in their serum [5].

Plausible, explanations for the different concentrations of these chemokines in the urine but not in the blood include differences in the rate of clearance of these chemokinases between Decliners and Non-decliners, as well as differences in sensitivity and detectability of the assays used. While the first possibility needs further study, the second possibility is unlikely considering the performance of the assays used in our study.

An alternative explanation for the findings reported by Wolkow et al. [5] is that kidney cells, primarily tubular, are the source of the elevated urinary concentrations of these chemokines. Although, the nature of the stimulus to synthesize these chemokines in tubular cells is unknown, it might originate from the glomerular filtrate. We hypothesize that impairment of the glomerular filtration barrier (evidenced by the presence of MA) permits injurious serum proteins or growth factors to leak into the urinary space. These putative factors, which we refer to as toxic urinary proteins (txUPs), may stimulate proximal tubular cells to secret chemokines/cytokines and other stress proteins indicating tubular damage that leads to tubular atrophy, interstitial fibrosis and early GFR loss. Recently it has been demonstrated in animal studies that tubular damage initiates a disease process that leads to inflammation, loss of blood vessels, interstitial fibrosis and glomerulosclerosis [6].

Here we report an *in vitro* study of the effects of urine on gene expression profiles in human proximal tubular cells (HK-2 cells). We postulate that exposure of HK-2 cells to urine from Decliners would induce a different gene expression profile than that induced by exposure to urine from Non-decliners due to the presence of putative txUPs in the former and their absence (or lower concentration) in the latter. The differentially expressed genes may reveal disease processes taking place in tubules during the development of early GFR decline in T1D.

Earlier expression studies include the analysis of candidate genes or candidate pathways (biased approach) in kidney tissue from diabetic rodent models and diabetic humans [7], [8]. Similarly, studies of a limited number of candidate genes and proteins have been conducted *in vitro* in HK-2 cells. Cells were exposed to urine from patients with severe proteinuria or urine from patients with focal glomerulosclerosis and patients with idiopathic minimal change disease, and the results were compared [9], [10]. In contrast, we used an unbiased genome-wide approach to characterize gene expression in our study of urines from patients with MA. All patients had normal renal function when their urine was collected, but during 10–12 years of follow-up, renal function became impaired in half and remained normal in the others. In this study we sought to identify differences in gene expression in HK-2 cells that might reflect exposure to putative txUPs present in the urine of patients who subsequently developed impaired renal function.

Recently, unbiased genome-wide transcriptome approaches have been applied to kidney tissue from patients with DN. Two studies catalogued gene expression profiles in microdissected human renal glomerula and tubules and interstitium obtained from kidneys with advanced DN and individuals who were healthy or diagnosed with minimal change disease. Gene expression was correlated with eGFR in the examined patients [11], [12]. These findings are compared with the results of our transcriptome analysis in HK-2 cells.

## Subjects and Methods

The Committee of Human Studies of the Joslin Diabetes Center approved the study, its protocols and informed consent procedures. Informed consent was obtained in writing from all participants involved in the study.

### Study design and study groups

This study examined *in vitro* effects of the putative txUPs on gene expression profiles in HK-2 cells using urine specimens obtained from two sets of patients; those who developed early eGFR decline during follow-up (Decliners) and those with stable eGFR during follow-up (Non-decliners).

All patients were Caucasian participants in the follow-up study titled “The Joslin Study on Natural History of Early Nephropathy in Type 1 Diabetes”. The urines for this project were obtained from patients who developed new onset MA and were subsequently followed for 8–12 years. Findings regarding changes in urinary albumin excretion and trajectories of renal function decline in these patients were reported by Perkins et al. and Merchant et al. [2], [13]. Urine specimens obtained from 79 of these patients were previously used in the proteomic study by Merchant et al. [13]

For the current study we identified 17 patients for whom we had urine specimens that met the following criteria. The specimen was obtained 2–5 years after onset of MA, and at least 10 ml of archived urine remained. Serial measurements of cystatin C in sera obtained during follow-up were used to trace the trajectory of estimated GFR. The patients were classified as Decliners and Non-decliners according to criteria described previously [3]. Briefly, patients with a GFR loss 3.3%/year or faster were considered Decliners. Patients with slower loss were considered Non-decliners.

Tests for contamination with endotoxins (Pyrogent Plus single test kit, Cambrex, Walkersville, MD) eliminated two specimens. From the remainder, we selected five from Decliners and five from Non-decliners whose characteristics were most similar (see Table 1). These urines were concentrated using columns with cutoff 5 kD (Millipore Amicon Ultra – 4, Billerica, MA).

**Table 1:** Clinical characteristics of patients at the time study-urine samples were obtained according to whether renal function subsequently declined or was stable during 8–10 year follow-up (data are medians and range).

	Decliners	Non-decliners
Men/Women (n/n)	4/1	4/1
Age at DM Dx (yrs)	12	14
Duration of DM (yrs)	21	24
HbA1c (%)	8.7	8.1
Urinary albumin (µg/ml)	61	49
cC-GFR (ml/min)	83	91
Rate of cC-GFR decline (%/yr)	3.5–12.1	0.5–2.0

cC-GFR estimated glomerular filtration rate based on serum cystatin C.

### Pooling urine specimens

To reduce the study cost, we pooled the concentrated urines from Decliners and Non-decliners into two samples, thereby reducing the number of RNA specimens to be processed from 60 to 12. The urine pools were added to serum-free K-SFM medium (Keratinocyte serum free medium - Invitrogen, GIBCO, Cat No. 17005-042) to give a final urinary albumin concentration that was 2.0 times that in the original samples. Glucose concentration was 25 mMol in the culture medium, which was changed every other day.

### *In vitro* Experiments

Cultured HK-2 cells (immortalized human PTC, 80% confluent) (ATCC, Manassas, VA) were exposed to K-SFM serum free medium combined with pooled urine from either Decliners or Non-decliners. Each experiment consisted of four cell cultures. Two were exposed to pooled urines from Decliners, one for 6 hours and one for 24 hours. Similarly, two were exposed to pooled urines from Non-decliners, one for 6 and one for 24 hours. The experiments were repeated three times several weeks apart.

After completion of all experiments, RNA was isolated from the HK-2 cells with RNeasy Micro Kit (Qiagen, Germantown, MD). The twelve specimens were analyzed for RNA quantity and normalized. cDNA was synthesized and hybridized with the Illumina Sentrix® Beadchip Array Human-6 (San Diego, CA) to obtain gene expression profiles. The chip had 24,000 probes that covered 19,600 genes. Array hybridization, washing, and scanning were performed by the core facility at the Enders Institute, Children's Hospital, Boston, MA according to Illumina-recommended protocols (San Diego, CA).

### Data Analysis

Raw expression data were analyzed with dChip software (www.dchip.org). Raw intensity values were normalized with an invariant set normalization method [14]. The criteria for identifying expression differences in the normalized data were: a fold change between Decliners and Non-decliners ≥1.3 (up-regulated) or ≤0.77 (down-regulated); an expression difference threshold for the absolute difference >100; and a P-value <0.05. The overlap between up-regulated genes in HK2 cells and up-regulated genes in biopsies was tested by a Fisher's exact test, and the overlap of down-regulated genes was tested similarly.

### Gene ontology analyses

Significantly regulated genes were analyzed for enrichment of gene ontology (GO) terms (molecular function and biological processes) using Genomatix pathway system software (http://www.genomatix.de). The report for each annotation included the observed and expected numbers of genes, the p value, and the total number of genes in the annotation. Because proteins frequently have different molecular functions and involvement in multiple biological processes, the same gene may be included in several different GO terms. To reduce redundancy (one gene present in many downstream terms) and gain an overview of general biology, significant GO terms for molecular function (MF) (p<0.01) were retrieved upstream towards its ancestors based on the GO Inferred Tree View (http://www.geneontology.org/, AMIGO version 1.8) in a manner similar to that described in DAVID: Database for Annotation, Visualization, and Integrated Discovery [15]. The different levels provided by Inferred Tree View allow users to annotate lists of genes at different levels of generality and specificity. Level 1 represents the most general categories and provides the most coverage, whereas Level 5 provides more specific information and less coverage. The GO vocabulary is a hierarchy, so a term at level 5 is a child of a term at level 1 for a given gene (note that for some genes there will be more than 5 levels). For a good balance between specificity and coverage, the terms presented in this analysis are at the fourth level downstream from the start of a molecular function (GO:0003674 molecular function (472694 gene products)). For biological processes (BP), a large number of terms passed the significance cut off (p<0.01), so only the 20 BP terms with the smallest P-values below 10^−6^ were selected for analysis. As was done for molecular function terms, BP terms are presented at the fourth level downstream from the start of a biological process (GO:0008150 biological process (443783 gene products)). In this report we considered only GO terms that had 5 or more genes observed.

The original files of significant GO terms with the most specific categories using terminal nodes are available upon request.

### Concordance between genes differentially expressed in HK-2 cells and genes differentially expressed in kidney biopsies of patients with DN

We compared our results in HK-2 cells with gene expression profiles in tubular and interstitial compartments of kidney biopsies obtained from patients with diabetes and various degrees of renal function impairment that were reported by Schmid et al. [11] and by Woroniecka et al. [12] deposited in Nephromine. The biopsies in Woroniecka's study were taken from 12 non-diabetic controls and 12 cases with advanced DN. The expression data were combined, and Spearman correlation coefficients were determined between gene expression and eGFR. The biopsies in Schmid's study were taken from 12 patients with advanced DN (proteinuria and a broad range of renal function impairment). Spearman correlation coefficients were determined between expression data and renal function. Both studies used Affymetrix arrays (n = 12,600 genes). Although the Illumina array used in the present study contained 19,600 genes, we used only the genes present on the Affimetrix array for comparison with Nephromine data.

## Results

### Study design and characteristics of the study group

Clinical characteristics of Decliners and Non-decliners are summarized in Table 1. At baseline, when urine specimens were obtained, the distributions of age at diagnosis of diabetes, duration of diabetes, and HbA1c were similar in both groups. Furthermore, estimated GFR (based on serum cystatin C) was normal in Decliners and Non-decliners and urinary albumin excretion was only moderately elevated. However, by design, Decliners and Non-decliners differed with regard to GFR change during follow-up. Decliners had significant GFR loss per year, whereas Non-decliners had minimal GFR changes. After 8–12 years of follow-up, average eGFR was 44 ml/min in Decliners and 82 ml/min in Non-decliners.

The urines were concentrated two fold and combined into a pooled urine from Decliners and a pooled urine from Non-decliners. The pools were added to serum-free tissue culture media in which HK-2 cells were grown. Two exposure times, 6 and 24 hours, were selected to increase the chance of detecting genes with different time-courses of expression during exposure. After completion of all experiments, HK-2 cells were collected and mRNA isolated. Genome-wide expression profiles were determined with the Illumina Sentrix® Beadchip Array Human-6.

### Gene expression profiles in HK-2 cells according to exposure time

**Figure 1:**
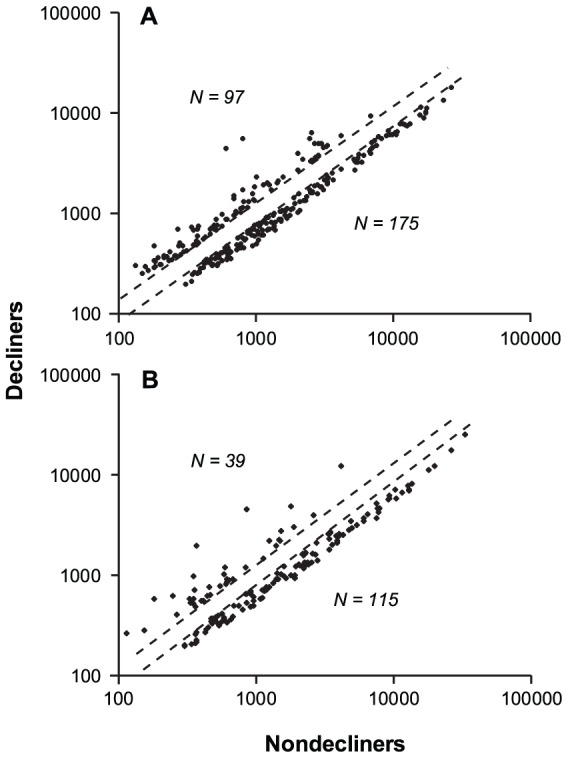
Plot of the gene expression level in HK2 cells exposed to urine from Decliners against the gene expression level in HK2 cells exposed to urine from Non-decliners. Broken diagonal lines represent fold changes of 1.3 and 0.77. **Panel A:** Expression levels after 6-hour exposure. **Panel B:** Expression levels after 24-hour exposure.

After normalization of the expression data, we applied pre-defined criteria to identify expression differences: a fold-change (≥1.3 or ≤0.77) and a nominal p-value (*P*<0.05). After 6-hour exposure 274 genes and after 24-hour exposure 154 genes were differentially expressed. Normalized expression of these genes is illustrated in Figure 1 with expression after exposed to Decliner urine plotted against that after exposure to Non-decliner urine. Of the genes differentially expressed after 6-hour of exposure, 99 were up-regulated and 175 down-regulated (Panel A). Similarly, of the genes differentially expressed after 24-hour exposure, 39 genes were up-regulated and 115 down-regulated (Panel B).

In total, 314 genes were differentially expressed at one or both exposure times. The distribution of these genes according to the temporal pattern of expression is shown in Table 2. Of the 119 up-regulated genes; 36 were up-regulated persistently (after both exposures), 67 were up-regulated temporarily (at 6-hours only), and 16 were up-regulated after a delay (at 24-hours only). Of the 195 down-regulate genes, 138 were down-regulated persistently, 45 genes were down-regulated temporarily, and 12 genes were down-regulated after a delay. All 314 are listed alphabetically in Table S1 according to temporal pattern of expression.

**Table 2:** Distribution of differentially expressed genes in HK2 according to their pattern of expression after 6-hour and 24-hour exposures cells to urine from Decliners or Non-decliners.

Pattern of expression:			
6-hr exposure	24-hr exposure	Number of genes	Numbers of genes correlated with eGFR in Nephromine
Up	Up	36	17 (47%)
Up	Normal	67	15 (22%)
Normal	Up	16	9 (56%)
Down	Down	138	13 (9%)
Down	Normal	45	6 (13%)
Normal	Down	12	3 (25%)
Total		314	63 (20%)

### GO analysis of genes differentially expressed in HK-2 cells

To identify GO terms (e.g. molecular function and biological processes) that are enriched in HK-2 cells in response to exposure to Decliner urine, we analyzed differentially expressed genes with Genomatix pathway system software (http://www.genomatix.de). Among the differentially expressed genes we found GO terms significantly enriched at level 5 for molecular function and biological processes. As described in Methods, to reduce redundancy (one gene present in many downstream terms) and gain an overview of general biology, the GO terms were retrieved upstream towards their ancestors up to level 4. For this analysis we considered GO terms that were significant at p<0.01 at level 5. Since there were hundreds of terms for biological processes significantly enriched at this level, we restricted analysis to the 20 terms with the lowest P-values (p<10^−6^).

Results of GO analysis for the 119 up-regulated genes are summarized in Tables 3 & 4. The two molecular function terms that were significantly enriched are described in more detail in the following. The first included genes that encode for receptor binding. Among all human genes, 1003 are classified under this term, and 22 of them were over-expressed in our study while only 6.95 were expected if differential expression had been by chance. It is interesting that among the 22 included genes, many encode chemokines and cytokines. The second enriched term included peptidase inhibitor activity. Out of a total of 163 human genes in this term, six were up-regulated while 1.13 were expected. With regard to GO terms for biological process the up-regulated genes were enriched in the following processes: defense response, response to other mechanisms, regulation of response to stress, regulation of response to stimulus and programmed cell death.

**Table 3:** Enriched GO molecular function terms for 119-up-regulated genes in HK-2 cells (Significantly enriched (P<0.01) GO terms were retrieved upstream towards its ancestors to level 4).

GO-term IDs	GO-term	# Genes observed	# Genes expected	# Genes total	List of observed genes
5102	Receptor binding	22	6.95	1003	*ADM, ADRB2, BID, C3, CCL2, CCL5, CCL20, CSF2, CXCL2, CXCL3, EBI3, HBEGF, ICAM1, IL6, IL8, JAK1, LYN, NAMPT, NEDD4, SAA1, SERPINE1, TNFSF10*
30414	Peptidase inhibitor activity	6	1.13	163	*C3, SERPINE1, SERPINB8, SLPI, TNFAIP3, WFDC2*

**Table 4:** Enriched GO biological process terms for 119 up-regulated genes in HK2 cells (top 20 significantly enriched BP terms (p<0.01) were retrieved upstream towards its ancestors to level 4).

GO Term Ids	GO Term	# Genes observed	# Genes expected	# Genes total	List of observed genes
6952	Defense response	29	6.72	910	*ADRB2, C3, CCL2, CCL20, CCL5, CD40, CXCL2, CXCL3, ELF3, ICAM1, IFNGR1, IL6, IL8, IRAK3, JAK1, LCN2, LYN, NFKB1, NFKBIA, PTX3, SAA1, SAA4, SERPINE1, STAT1, STAT5A, TFRC, TNFAIP3, TNFRSF1B, TNIP1*
51707	Response to other organism	17	3.23	437	*ADM, CCL2, CCL5, CCL20, CXCL2, ICAM1, IFNGR1, IL6, IL8, IRAK3, LCN2, NFKBIA, PIM2, PTX3, SERPINE1, STAT1, TNFAIP3*
50794	Regulation of cellular process	65	48.73	6593	*ADM, ADRB2, ARF4, BCL2A1, BID, BIK, BIRC3, C3, CCL2, CCL20, CCL5, CCNE2, CD40, CNIH, CSF2, DDR1, DNAJB6, EBI3, EIF4B, ELF3, FNDC3B, FOSL2, HBEGF, HSP90AA1, ICAM1, IFNGR1, IL6, IL8, IRAK3, JAK1, LCN2, LGR4, LYN, MAPK6, MIER1, MMP7, NAB1, NAMPT, NAP1L1, NBN, NEDD4, NFKB1, NFKBIA, PIM2 PLAT, PTX3, RAC2, RCAN1, SAA1, SAT1, SDC4, SERPINE1, SH3BP1, SLC11A2, SOD2, SRPK1, ST5, STAT1, STAT5A, TNFAIP3, TNFRSF1B, TNFRSF9, TNFSF10, TRAF1, TRIM16*
80134	Regulation of response to stress	18	3.62	490	*ADRB2, C3, CCL5, HBEGF, IFNGR1, IL6, IRAK3, JAK1, LYN, NFKB1, NFKBIA, PLAT, SAA1, SERPINE1, STAT1, STAT5A, TNFAIP3, TNFRSF1B*
48583	Regulation of response to stimulus	30	13.07	1769	*ADRB2, C3, CCL5, CD40, CSF2, HBEGF, ICAM1, IFNGR1, IL6, IL8, IRAK3, JAK1, LYN, MIER1, NEDD4, NFKB1, NFKBIA, PIM2, PLAT, RAC2, SAA1, SERPINE1, ST5, STAT1, STAT5A, TNFAIP3, TNFRSF1B, TNFSF10, TRAF1, TRIM16*
12501	Programmed cell death	29	9.78	1323	*ADM, ADRB2, BCL2A1, BID, BIK, BIRC3, CCL2, CCL5, CD40, CSF2, DNAJB6, IL6, LCN2, NFKB1, NFKBIA, PIM2, RNF144B, SERPINE1, SLC11A2, SOD2, STAT1, STAT5A, TJP2, TNFAIP3, TNFRSF1B, TNFRSF9, TNFSF10, TRAF1, ZC3H12A*

Results of GO analysis for the down-regulated genes are summarized in Tables 5 & 6. These genes were disproportionately represented in GO terms for molecular functions such as cation binding, hydrolase activity acting on ester bonds, and DNA binding. With regard to GO terms for biological process, the down-regulated genes were disproportionately represented in the following processes: regulation of metabolic processes, nucleic acid metabolic processes, cellular macromolecule metabolic processes, cellular response to stress and macromolecule biosynthetic processes.

**Table 5:** Enriched GO molecular function terms for 195 down-regulated genes in HK-2 cells (Significantly enriched (P<0.01) GO terms were retrieved upstream towards its ancestors to level 4).

GO-term Ids	GO-term	# Genes observed	# Genes expected	# Genes total	List of observed genes
43169	Cation binding	36	30.26	**3888**	*AIRE, APOBEC3A, CXXC5, DBF4, ERAP2, MICAL3, MSRB3, OVOL2, PDLIM5, PTGS2, PXN, SMAP1, SUV39H2, TRIM58, TRIM74, TTF2, XIAP, XPNPEP3, ZMAT3, ZNF14, ZNF33A, ZNF417, ZNF430, ZNF454, ZNF542, ZNF549, ZNF608, ZNF620, ZNF623, ZNF652, ZNF665, ZNF667, ZNF786, ZNF791, ZNF91, ZNF98*
16788	Hydrolase activity, acting on ester bonds	15	6.84	717	*ARSG, ARSK, CDC14B, CDC25B, CTDSPL, DUSP1, DUSP19, DUSP5, EXOSC2, N4BP2, NT5C2, OLAH, PNPLA8, PPME1, USP14*
3677	DNA binding	35	21.84	2287	*AHR, AIRE, BLZF1, CREB1, CXXC5, DMC1, FOXL2, GABPB2, GTF3C6, LRRFIP1, MCM8, MITF, MSH3, NAT14, NFAT5, NFATC3, OVOL2, RAD51L1, TTF2, ZNF14, ZNF33A, ZNF417, ZNF430, ZNF454, ZNF542, ZNF549, ZNF620, ZNF623, ZNF652, ZNF665, ZNF667, ZNF786, ZNF791, ZNF91, ZNF98*

**Table 6:** Enriched GO biological process terms for 194 down-regulated genes in HK2 cells (top 20 significantly enriched BP terms (P<0.01) were retrieved upstream towards its ancestors to level 4).

GO Term IDs	GO Term	# Genes observed	# Genes expected	# Genes total	List of observed genes
19222	Regulation of metabolic process	47	33.46	3691	*AHR, AIRE, ATG10, BLZF1, CDC14B, CDC25B, CREB1, DLC1, DUSP1, DUSP19, DUSP5, FOXL2, GABPB2, HIPK2, ID1, ID2, ITCH, ITGB3, KCNH6, LRRFIP1, MAP3K9, MITF, MSH3, NFAT5, NFATC3, NPR1, OVOL2, PCSK9, PTGS2, PXN, SMAP1, SYDE2, TADA3, USP14, ZNF14, ZNF33A, ZNF417, ZNF430, ZNF454, ZNF542, ZNF549, ZNF620, ZNF667, ZNF786, ZNF791, ZNF91, ZNF98*
6139	Nucleobase-containing compound metabolic process	47	38.00	4192	*AHR, AIRE, BLZF1, C9orf80, CDC14B, CREB1, DBF4, DMC1, EXOSC2, FOXL2, GABPB2, GTF3C6, HIPK2, ID1, ID2, INTS3, ITCH, KCNH6, LRRFIP1, MCM8, MITF, MSH3, NAT14, NFAT5, NFATC3, OVOL2, RAD51L1, RPL21, RPSA, SMG1, SRRM2, TADA3, TEP1, TTF2, ZNF14, ZNF33A, ZNF417, ZNF430, ZNF454, ZNF542, ZNF549, ZNF620, ZNF667, ZNF786, ZNF791, ZNF91, ZNF98*
90304	Nucleic acid metabolic process	35	31.27	3449	*AHR, AIRE, BLZF1, CREB1, FOXL2, GABPB2, GTF3C6, HIPK2, ID1, ID2, ITCH, KCNH6, LRRFIP1, MITF, NAT14, NFAT5, NFATC3, OVOL2, RPL21, RPSA, TADA3, TTF2, ZNF14, ZNF33A, ZNF417, ZNF430, ZNF454, ZNF542, ZNF549, ZNF620, ZNF667, ZNF786, ZNF791, ZNF91, ZNF98*
44260	Cellular macromolecule metabolic process	67	50.93	5618	*AHR, AIRE, ATG10, BLZF1, C9orf80, CDC14B, CDC25B, CREB1, DBF4, DLC1, DMC1, DUSP1, DUSP19, DUSP5, EXOSC2, FOXL2, FUT6, GABPB2, GNPNAT1, GTF3C6, HIPK2, ID1, ID2, INTS3, ITCH, ITGB3, KCNH6, LRRFIP1, MAP3K9, MCM8, MITF, MRP63, MSH3, MSRB3, NAT14, NFAT5, NFATC3, NPR1, OVOL2, PCSK9, PPME1, PXN, RAD51L1, RPL21, RPSA, SMG1, SRRM2, SUV39H2, TADA3, TEP1, TTF2, TTLL12, USP14, WDR82, ZNF14, ZNF33A, ZNF417, ZNF430, ZNF454, ZNF542, ZNF549, ZNF620, ZNF667, ZNF786, ZNF791, ZNF91, ZNF98*
33554	Cellular response to stress	16	7.77	857	*ATG10, C9orf80, CDC14B, CREB1, DUSP19, HIPK2, INTS3, ITCH, MAP3K9, MSH3, PCSK9, PXN, RAD51L1, SMG1, XIAP, ZMAT3*
9059	Macromolecule biosynthetic process	42	29.01	3200	*AHR, AIRE, ATG10, BLZF1, CREB1, DBF4, FOXL2, FUT6, GABPB2, GNPNAT1, GTF3C6, HIPK2, ID1, ID2, ITCH, ITGB3, KCNH6, LRRFIP1, MCM8, MITF, MRP63, NAT14, NFAT5, NFATC3, OVOL2, RPL21, RPSA, TADA3, TTF2, ZNF14, ZNF33A, ZNF417, ZNF430, ZNF454, ZNF542, ZNF549, ZNF620, ZNF667, ZNF786, ZNF791, ZNF91, ZNF98*

### Overlap between genes differentially expressed in HK-2 cells and in tubulointerstitial fraction obtained from biopsies of human kidney with advanced DN

**Figure 2:**
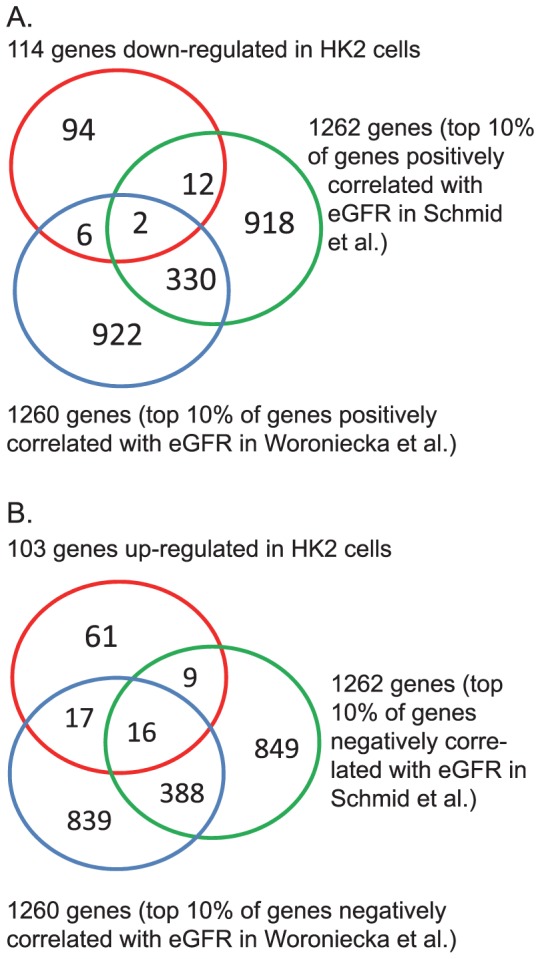
Overlap of the set of differentially regulated genes in HK-2 cells in response to urines from Decliners and Non-decliners and the corresponding sets of genes in tubular and interstitial compartments of kidney biopsies obtained from patients with advanced DN. **Panel A:** Down-regulated genes in HK-2 cells and in tubular and interstitial compartments of kidney biopsies (P-value  = 0.34 for overlap with data of Woroniecka et al. [12] and 0.43 for overlap with data of Schmid et al. [11]). **Panel B:** Up-regulated genes in HK-2 cells and in tubular and interstitial compartments of kidney biopsies (P-value <10^−9^ for overlap with data of Woroniecka et al. [12] and <10^−4^ for overlap with data of Schmid et al. [11]).

An additional goal of our study was to determine the extent of overlap between [1] disease processes (as reflected in gene expression) that were initiated in our *in vitro* study of putative txUPs in urines of patients who subsequently developed renal function decline and [2] the disease processes observed in tubules and interstitium in biopsies from kidneys of patients with DN. Toward that end, we used the findings reported by Woroniecka's et al. and Schmid et al., both of whom studied the same Affymetrix subset of 12,600 genes [11], [12]. For this comparison, we restricted our Illumina dataset of 19,600 genes to that same subset. This reduced the available number of down-regulated or up-regulated genes in our dataset from 195 to 114 and 119 to 103, respectively. The overlaps among the three datasets are illustrated with a Venn diagram (Figure 2). For the biopsy studies, the investigators related a gene's expression in tubular and interstitial compartments of kidney biopsies obtained from patients with advanced DN to that patient's eGFR using the Spearman correlation coefficient. A positive correlation (low expression together with low eGFR and high expression with high eGFR) was interpreted as linking eGFR loss with down-regulation of gene expression. A negative correlation (high expression together with low eGFR and low expression with high eGFR) was interpreted as linking eGFR loss with up-regulation.

The 10% of genes with the strongest positive correlations with eGFR were deemed down-regulated: 1260 in the data of Woroniecka et al. [12] and 1262 in the data of Schmid et al. [11]. Overlap of these two subsets with each other and with the 114 genes down-regulated in HK-2 cells by exposure to urine from Decliners is shown in Figure 2A. Of the down-regulated genes in HK-2 cells, only eight were common to the Woroniecka et al. subset, and fourteen to the Schmid et al. subset. Overlaps of 11.4 genes were expected due to chance; thus, neither was statistically significant. Down-regulated genes in HK-2 cells were not considered further.

The 10% of genes with the strongest negative correlations with eGFR were deemed up-regulated: 1260 in the data of Woroniecka et al. [12] and 1262 in the data of Schmid et al. [11]. Overlap of these two subsets with each other and with the 103 genes up-regulated in HK-2 cells by exposure to urine from Decliners is shown in Figure 2B. Out of the 103 up-regulated genes in HK-2 cells, 33 were common to the Woroniecka et al. subset, and 25 to the Schmid et al. subset. For each comparison, only 10.3 genes were expected by chance. Thus, there are significant excesses in the overlaps between up-regulated genes in HK-2 cells exposed to urine from Decliners and subsets of up-regulated genes in the datasets of Woroniecka et al. and Schmid et al. (p<10^−9^ and p<10^−4^ respectively).

The up-regulated genes in HK-2 cells that overlap with the biopsy subsets of genes are listed in Table 7. The pattern of expression of these genes according to the duration of exposure to urines varied. Over-expression was persistent for 17, temporary for 15 (at 6 hours only), and delayed for 9 (at 24 hours only). Regardless of these different temporal patterns, all of these genes were over-expressed in tubular and interstitial compartments of kidney biopsies obtained from patients with advanced DN, and the level of their over-expression was strongly but negatively correlated with eGFR in patients at the time of biopsies (Spearman correlation coefficients varied between −0.82 and −0.44). These up-regulated and over-lapping genes were subjected to GO analysis. The results of the analysis for molecular functions were similar to the findings reported in Table 3 for all 119 up-regulated genes (data not shown). The results of the analysis for biological processes were different and are shown in Table 8. The first two GO terms defense response and response to other organism are similar as in Table 4. The other significantly enriched processes were different and included response to organic substance, response to drug, cellular response to biologic stimulus, regulation of cellular processes, innate immune response, and protein metabolic process.

**Table 7:** List of 41 up-regulated genes overlapping between: A) up-regulated genes in HK-2 cells exposed to urines from Decliners and B) up-regulated genes in tubular and interstitial compartment of kidney biopsies obtained from patients with advanced diabetic nephropathy (genes strongly negatively correlated with eGFR).

		A) Findings in HK-2 cells:				B) Findings in tubular/interstitial compartment:			
Gene Name	Entrez ID	Fold change	(p)	Fold change	(p)	Woroniecka's datasetSpearman Correlation		Schmid's datasetSpearman Correlation	
		6 hrs expos.		24 hrs expos.		(r)	(p)	(r)	(p)
*Genes up-regulated during both exposures*									
*ADRB2*	154	2.13	0.009	1.36	0.002	−0.77	0.00		
*BIRC3*	330	1.40	0.126	1.77	0.019	−0.67	0.00	−0.68	0.01
*CCL2*	6347	2.27	0.011	1.98	8xE-04	−0.70	0.00		
*CCL5*	6352	2.24	0.001	1.83	4xE-05	−0.76	0.00		
*IL8*	3576	7.23	0.006	3.23	0.004	−0.59	0.00		
*JAK1*	3716	1.75	0.035	1.73	0.017	−0.49	0.01		
*LOX*	4015	1.43	0.050	1.67	0.041			−0.60	0.03
*LCN2*	3934	1.87	0.002	5.20	1xE-04	−0.59	0.00		
*NAMPT*	10135	1.51	0.004	1.33	0.085			−0.49	0.06
*NBN*	4683	1.34	0.017	1.39	0.027	−0.44	0.02	−0.46	0.08
*PTX3*	5806	1.86	0.009	1.31	0.002			−0.51	0.06
*SLPI*	6590	1.76	0.004	2.76	9xE-04	−0.69	0.00	−0.46	0.08
*SOD2*	6648	1.69	0.003	1.84	0.001	−0.74	0.00		
*ST5*	6764	1.92	0.022	1.31	0.005	−0.75	0.00		
*TFRC*	7037	1.47	0.034	1.73	0.002			−0.49	0.06
*TNFAIP2*	7127	2.51	0.004	1.45	0.005	−0.80	0.00		
*TNFRSF1B*	7133	1.84	0.009	1.36	0.013	−0.68	0.00		
*Genes up-regulated during 6 hrs. exposure:*									
*BCL2A1*	597	2.59	0.005	1.17	0.024	−0.62	0.00		
*DDR1*	780	1.31	0.015	1.13	0.173	−0.58	0.00	−0.51	0.05
*DSE*	29940	1.82	0.031	1.25	0.155	−0.59	0.00	−0.55	0.04
*FOSL2*	2355	1.50	0.010	1.25	0.004			−0.51	0.05
*ICAM1*	3383	1.51	0.003	1.20	0.103	−0.61	0.00	−0.47	0.07
*KIF2A*	3796	1.34	0.026	1.09	0.015	−0.53	0.01	−0.65	0.01
*LAMB3*	3914	1.84	0.046	1.28	0.006	−0.55	0.00		
*LYN*	4067	1.47	0.031	1.14	0.131	−0.80	0.00		
*MYO1B*	4430	1.39	0.042	1.25	0.010	−0.50	0.01	−0.55	0.04
*NAP1L1*	4673	1.46	0.030	1.13	0.601	−0.69	0.00	−0.46	0.07
*NFKB1*	4790	1.77	0.013	1.17	0.024			−0.71	0.01
*RAC2*	5880	1.34	0.008	1.20	0.017	−0.75	0.00		
*SERPINB8*	5271	1.58	0.036	0.99	0.826			−0.50	0.06
*SLC43A3*	29015	1.36	0.007	1.11	0.131	−0.56	0.00		
*TRAM1*	23471	1.32	0.017	1.10	0.113	−0.50	0.01	−0.49	0.06
*Genes up-regulated during 24 hrs. exposure.*									
*C3*	718	1.27	0.075	1.60	0.011	−0.78	0.00	−0.60	0.02
*DHX15*	1665	1.23	0.045	1.34	0.007			−0.60	0.03
*ELF3*	1999	1.07	0.446	1.58	0.006	−0.50	0.01	−0.53	0.05
*LAMC2*	3918	1.03	0.835	1.34	0.016	−0.75	0.00	−0.50	0.06
*MMP7*	4316	1.27	0.045	1.99	3xE-05	−0.82	0.00	−0.48	0.07
*TMEM132A*	54972	1.17	0.012	1.31	0.007	−0.62	0.00		
*TNFSF10*	8743	1.10	0.299	1.47	0.007	−0.82	0.00	−0.75	0.00
*STAT1*	6772	0.89	0.276	1.43	0.003	−0.70	0.00	−0.54	0.04
*WFDC2*	10406	1.12	0.197	1.31	0.019	−0.79	0.00	−0.65	0.02

**Table 8:** Overlapping gene: Enriched GO Biological process terms for up-regulated genes in HK2 cells (top 20 enriched BP terms (P<0.01) were retrieved upstream towards its ancestors to level 4).

GO Term-ID	GO Term	# Genes observed	# Genes expected	# Genes total	List of observed genes
6952	defense response	15	2.54	910	*ADRB2, C3, CCL2, CCL5, ELF3, ICAM1, IL8, JAK1, LCN2, LYN, NFKB1, PTX3, STAT1, TFRC, TNFRSF1B*
51707	response to other organism	6	1.22	437	*CCL2, CCL5, ICAM1, IL8, LCN2, STAT1*
10033	response to organic substance	12	3.37	1209	*CCL2, CCL5, DDR1, ICAM1, IL8, JAK1, LCN2, LOX, LYN, STAT1, TFRC, TNFRSF1B*
42493	response to drug	8	0.80	288	*CCL2, CCL5, ICAM1, LCN2, LOX, LYN, NBN, STAT1*
71216	cellular response to biotic stimulus	5	0.21	77	*CCL2, ICAM1, IL8, LCN2, STAT1*
50794*	regulation of cellular process	12	NA	6593	*ADRB2, CCL2, CCL5, DDR1, FOSL2, IL8, LYN, MMP7, NAP1L1, NBN, SOD2, STAT1*
45087	innate immune response	8	1.17	418	*C3, CCL2, CCL5, ICAM1, JAK1, LCN2, NFKB1, STAT1*
19538	protein metabolic process	5	0.33	119	*CCL5, DDR1, JAK1, LYN, STAT1*

* denotes GO term by itself is not significantly enriched, but some of its downstream terms is significantly enriched.

## Discussion

The risk of early GFR decline in T1D patients with MA is associated with elevated concentrations of several urinary chemokines, such as IL-6, IL-8, MCP-1(CCL2), IP-10 (CXCL10) and MIP-1δ (CCL15) [5]. Because the concentrations are not elevated in serum, we postulate that kidney cells, primarily tubular, are the source of the urinary elevations. The nature of the stimulus, however, is unknown. We hypothesized that minimal impairment of the glomerular filtration barrier (evidenced by the presence of MA) allows certain serum proteins or growth factors to leak into the urinary space where, in contrast to the vasculature, they may be toxic to proximal tubular cells and cause damage that results in synthesis of the elevates chemokines. Furthermore, these putative toxic urinary proteins (txUPs) should be present specifically in those patients with MA in whom renal function decline subsequently develops.

Results of this study in HK-2 cells support our hypothesis. Following exposure to the urine of Decliners whose renal function later declined, expression of 314 genes differs from their expression following exposure to urine from Non-decliners whose function remained stable. Urine from Decliners led to up-regulation of 119 and down-regulation of 195 genes. In a GO analysis of the up-regulated genes, two molecular function terms are significantly enriched. One includes receptor binding terms, and the second includes peptidase inhibitor activity. With regard to GO terms for biological processes, up-regulated genes are enriched for the following processes: defense response, response to other mechanisms, regulation of response to stress, regulation of response to stimulus and programmed cell death. It is interesting that up-regulated genes include some that encoded for chemokines, including those reported by Wolkow et al. [5], *CCL2 (MCP-1), IL-6, and IL-8*, and others such as *CCL5 (RANTES)*, *CCL20 (MIP-3a)*, *CSF2 (GMCSF)*, *CXCL2 (MIP-2a)*, and *CXCL3 (MIP-2b)* that were not examined by Wolkow et al. Over-expression of these genes in HK-2 cells is a coordinated defense response to other organisms or to stress and is part of programmed cell death. It most likely accounts for elevated levels of these chemokines in urine.

GO analysis of the down-regulated genes assigns them to molecular functions such as cation binding, hydrolase activity acting on ester bonds, and DNA binding. GO terms for biological processes assigns the down-regulated genes to the regulation of metabolic processes, nucleic acid metabolic processes, cellular macromolecule metabolic processes, cellular response to stress and macromolecule biosynthetic processes. Since all these genes were under-expressed one can infer that all the above processes are suppressed in HK-2 cells in response to urines from Decliners.

An important goal of our study was to determine the extent of overlap between the expression profiles in HK-2 cells exposed to urine from Decliners and expression profiles in kidney tissue (tubules and interstitium) of patients with advanced DN and significantly impaired renal function [11], [12]. Among the genes up-regulated in tubules and interstitium in advanced DN as reported by Schmid et al. and Woroniecka et al., 42 overlapped with up-regulated genes in HK-2 cells, well beyond the number expected by chance. This similarity indicates that certain disease processes in tubules and interstitium present in advanced DN have already begun when patients have minimal MA and normal renal function. Quite plausibly, these processes were induced by the putative txUPs.

What is the nature of txUPs? Many authors have investigated whether the putative txUPs might be the high concentration of albumin present in patients with overt proteinuria. Based on *in vitro* studies, two processes are proposed to initiate tubulointerstitial disease. Enhanced protein intake by proximal tubule cells results in lysosomal rupture and direct tubule toxicity and stimulates synthesis of cytokine and chemokines that enhance the inflammatory response and activate fibrotic processes in the interstitial compartment [16], [17]. These studies, however, suffered from many methodological problems, including use of extremely high concentrations of albumin. Recently Bains and Brunskill [18], [19] reviewed these studies and deemed them inconclusive.

In a just published review Bonventre proposes the hypothesis that persistent tubular damage initiates inflammation of tubules and interstitium, loss of blood vessels and interstitial fibrosis and results in glomerulosclerosis that may lead to renal function decline [20]. He suggested several possible mechanisms or exposures that may be responsible for the injury to proximal tubules in diabetes. Whereas some of them, for example excess of reactive oxygen species (ROS), are related to abnormal metabolism of glucose in proximal tubule cells per se, the others might be considered candidates for txUPs. For example, TGF-B1 that is produced by mesangial cells in the presence of hyperglycemia may be excreted into the urine. As a pro-fibrotic factor, it may affect proximal tubules and interstitium. Also, chronic hyperglycemia results in nonenzymatic glycation of protein and formation of advanced glycation end-products (AGEs). These are excreted into the urine and reabsorbed in proximal tubules where they undergo catabolism. This may result in production of IL-6, IL-8, MCP-1, CTGF, TGF-B1 and VEGF. Note that the genes for the first three of these chemokines were up-regulated in our study, and genes for the remaining three were not.

Several chemokines/cytokines are excreted in excess in urine in patients with established DN [5], [21]. However, until the study by Wolkow et al. of patients with MA, the ability of chemokines, such as IL-6, IL-8, MCP-1, IP-10 and MIP-1δ, to predict the future occurrence of early renal function decline was not recognized. As noted above, several of these chemokines are up-regulated in the HK-2 cells by exposure to urine from Decliners. Since the chemokines in the urine cannot up-regulate expression of their genes in tubules, these chemokines are not the putative txUPs, and some other stimulus must be involved. A cytokine that has been implicated in the development of DN, tumor necrosis factor-alpha (TNFα), could plausibly be that stimulus [22]. Interestingly, many of the up-regulated genes in our HK-2 cells are up-regulated by in vitro exposure of renal epithelial cells to TNFα [23], [24].

TNFα is a pleiotropic cytokine that plays an essential role in mediating inflammatory processes [25]. TNF is recognized by two receptors, TNFR1 and TNFR2. In plasma, TNFα appears as free or bound to its receptors. Hasegawa et al. were the first to implicate TNFα in the pathogenesis of DN [22]. Recently we demonstrated that circulated TNFα and its two receptors were the strongest predictors of progression to CKD3 in Type 1 diabetes and ESRD in Type 2 diabetes [4], [26]. Although the effects of the receptors were stronger than TNFα, one cannot exclude the later as a causal factor due to significant correlation among these three markers. This difficulty is compounded by measurement problems of TNFα as free or bound in plasma and serum [26]. Moreover, its concentration in urine is very low, and we were unable to measure it reliably (data not shown).

Finally, we ought to acknowledge the strengths and possible shortcomings of our study. First, application of global transcriptome analysis, rather than an analysis of selected pathways (biased approach), is a strength. This strength, however, was somewhat compromised in the search for overlap of our set of up- or down-regulated pathways in HK-2 cells with the corresponding sets of genes in tubular/interstitial compartments of kidney biopsies previously reported [11], [12]. The Affymetrix platform used in those studies included only 61% of the genes represented on our Illumina platform (12,000 out of 19,600). How much overlap was missed because of this difference is unknown. Despite this shortcoming, the significant overlap of the set of up-regulated genes in our HK-2 cells and the sets in two independent biopsy studies of diabetic kidneys is a strong validation/replication of our findings. Second, we used pooled urines from Decliners and Non-decliners to reduce cost and minimize the difficulty of maintaining standardized conditions across all experiments, which would be large number if we examined each patient individually. This element of the study design is a strength for it most likely increased the study's sensitivity. Third, our use of a two-fold concentration of urine may be seen as a shortcoming. Possibly the concentrations of the putative txUPs were too low for all relevant genes to be differentially expressed in our experiments. Our choice was arbitrary and, to offset this possibility, we performed parallel studies with exposures of 6 and 24 hours. Fourth, a strength that distinguishes our study from all other *in vitro* studies was that the cultured cells were exposed to serum-free medium. This reduces exposure of cells to serum growth factors and high concentration of albumin present in serum. Note: the media contained high levels of glucose. Fifth, we used immortalized tubular epithelial cell line HK-2 in our experiments. It is unknown whether the findings would be the same if primary cultures of tubular epithelial cells had been used.

## Supporting Information

Table S1 List of up-regulated and down regulated genes in HK-2 cells exposed to urines from Decliners and gene differentially expressed in tubular and interstitial compartment of kidney biopsies obtained from patients with advanced diabetic nephropathy and reported recently (11,12).
(DOC)
