# Supplementary material for: Transcriptome Analysis of Proximal Tubular Cells (HK-2) Exposed to Urines of Type 1 Diabetes Patients at Risk of Early Progressive Renal Function Decline
Source: PLoS One. 2013 Mar 7;8(3):e57751. doi: 10.1371/journal.pone.0057751 (PMC3591403; doi:10.1371/journal.pone.0057751)
Supplement: Table S1 — List of up-regulated and down regulated genes in HK-2 cells exposed to urines from Decliners and gene differentially expressed in tubular and interstitial compartment of kidney biopsies obtained from patients with advanced diabetic nephropathy and reported recently (11,12). (DOC) [file pone.0057751.s001.doc]

Table S1: List of up-regulated and down regulated genes in HK-2 cells exposed to urines from Decliners and gene differentially expressed in tubular and interstitial compartment of kidney biopsies obtained from patients with advanced diabetic nephropathy and reported recently (11,12).

_________________________________________________________________________________________________________

Findings in HK-2 cells: Findings in tubular/interstitial compartment

Woroniecka’s dataset (12) Schmid’s dataset (11)

Gene Name Entrez ID Fold change (p) Fold change (p), Spearman Spearman

6 hrs expos. 24 hrs expos. Correlation (r) (p) Correlation (r) (p)

*Genes up-regulated during both exposures*

| ADAMTS9 | 56999 | 1.43 | 0.016 | 1.38 | 9xE-05 |  |  |  |  |
| --- | --- | --- | --- | --- | --- | --- | --- | --- | --- |
| *ADRB2* | 154 | 2.13 | 0.009 | 1.36 | 0.002 | -0.77 | 0 |  |  |
| *BIRC3* | 330 | 1.4 | 0.126 | 1.77 | 0.019 | -0.67 | 0 | -0.68 | 0.01 |
| *CCL2* | 6347 | 2.27 | 0.011 | 1.98 | 8xE-04 | -0.7 | 0 |  |  |
| *CCL20* | 6364 | 6.96 | 0.002 | 5.36 | 0.002 |  |  |  |  |
| *CCL5* | 6352 | 2.24 | 0.001 | 1.83 | 4xE-05 | -0.76 | 0 |  |  |
| *CD40* | 958 | 1.44 | 0.002 | 1.3 | 0.034 |  |  |  |  |
| *CSF2* | 1437 | 2.15 | 0 | 2.3 | 0.03 |  |  |  |  |
| *CXCL2* | 2920 | 2.56 | 0.012 | 1.54 | 0.031 |  |  |  |  |
| *EIF4B* | 1975 | 1.3 | 0.076 | 1.38 | 0.05 |  |  |  |  |
| *HAS3* | 3038 | 1.51 | 0.003 | 1.33 | 0.024 |  |  |  |  |
| *IL4I1* | 259307 | 1.57 | 0 | 1.55 | 0.016 |  |  |  |  |
| *IL6* | 3569 | 2.15 | 0.015 | 1.5 | 0.005 |  |  |  |  |
| *IL8* | 3576 | 7.23 | 0.006 | 3.23 | 0.004 | -0.59 | 0 |  |  |
| *JAK1* | 3716 | 1.75 | 0.035 | 1.73 | 0.017 | -0.49 | 0.01 |  |  |
| *LCN2* | 3934 | 1.87 | 0.002 | 5.2 | 1xE-04 | -0.59 | 0 |  |  |
| *LOX* | 4015 | 1.43 | 0.069 | 1.67 | 0.041 |  |  | -0.6 | 0.03 |
| *NAMPT* | 10135 | 1.51 | 0.004 | 1.33 | 0.085 |  |  | -0.49 | 0.06 |
| *NBN* | 4683 | 1.34 | 0.017 | 1.39 | 0.027 | -0.44 | 0.02 | -0.46 | 0.08 |
| *NEDD4* | 4734 | 1.49 | 0.002 | 1.32 | 0.006 |  |  |  |  |
| *PLAT* | 5327 | 1.49 | 0.009 | 1.42 | 0.036 |  |  |  |  |
| *PTX3* | 5806 | 1.86 | 0.009 | 1.31 | 0.002 |  |  | -0.51 | 0.06 |
| *RNF144B* | 255488 | 2.6 | 0.018 | 1.56 | 0.003 |  |  |  |  |
| *SAA1* | 6288 | 1.43 | 0.002 | 2.92 | 6xE-05 |  |  |  |  |
| *SAA2* | 6289 | 1.39 | 0.013 | 2.71 | 0.011 |  |  |  |  |
| *SAA4* | 6291 | 1.79 | 0 | 2.47 | 9xE-05 |  |  |  |  |
| *SLC11A2* | 4891 | 2.02 | 0.002 | 1.36 | 0.019 |  |  |  |  |
| *SLPI* | 6590 | 1.76 | 0.004 | 2.76 | 9xE-04 | -0.69 | 0 | -0.46 | 0.08 |
| *SOD2* | 6648 | 1.69 | 0.003 | 1.84 | 0.001 | -0.74 | 0 |  |  |
| *ST5* | 6764 | 1.92 | 0.022 | 1.31 | 0.005 | -0.75 | 0 |  |  |
| *TFRC* | 7037 | 1.47 | 0.034 | 1.73 | 0.002 |  |  | -0.49 | 0.06 |
| *TNFAIP2* | 7127 | 2.51 | 0.004 | 1.45 | 0.005 | -0.8 | 0 |  |  |
| *TNFRSF1B* | 7133 | 1.84 | 0.009 | 1.36 | 0.013 | -0.68 | 0 |  |  |
| *TNFRSF9* | 3604 | 1.84 | 0.001 | 1.35 | 0.018 |  |  |  |  |
| *TNIP1* | 10318 | 2.27 | 0.007 | 1.3 | 0.008 |  |  |  |  |
| *TRAF1* | 7185 | 1.65 | 0.006 | 1.35 | 0.009 |  |  |  |  |
| *Genes up-regulated during 6hrs. exposure* | | | | | |  |  |  |  |
| ABCA12 | 26154 | 1.4 | 0.001 | 1.23 | 0.075 |  |  |  |  |
| *ADM* | 133 | 1.51 | 0.001 | 1.29 | 0.036 |  |  |  |  |
| *ARF4* | 378 | 1.31 | 0.015 | 0.92 | 0.446 |  |  |  |  |
| *ATP1B1* | 481 | 1.66 | 0.016 | 1.06 | 0.501 |  |  |  |  |
| *BCL2A1* | 597 | 2.59 | 0.005 | 1.17 | 0.024 | -0.62 | 0 |  |  |
| *BID* | 637 | 1.34 | 0.031 | 1.09 | 0.285 |  |  |  |  |
| *BIK* | 638 | 1.37 | 0.018 | 1.24 | 0.051 |  |  |  |  |
| *CAAP1* | 79886 | 1.3 | 0.023 | 1 | 0.953 |  |  |  |  |
| *CCNE2* | 9134 | 1.41 | 0.002 | 1.15 | 0.15 |  |  |  |  |
| *CDV3* | 55573 | 1.45 | 0.003 | 1.1 | 0.41 |  |  |  |  |
| *CLDN12* | 9069 | 1.4 | 0.029 | 1.09 | 0.073 |  |  |  |  |
| *CXCL3* | 2921 | 1.67 | 0.008 | 1.18 | 0.213 |  |  |  |  |
| *DDR1* | 780 | 1.31 | 0.015 | 1.13 | 0.173 | -0.58 | 0 | -0.51 | 0.05 |
| *DIEXF* | 27042 | 1.35 | 0.033 | 1.14 | 0.032 |  |  |  |  |
| *DNAJB6* | 10049 | 1.33 | 0.04 | 1.21 | 0.107 |  |  |  |  |
| *DSE* | 29940 | 1.82 | 0.031 | 1.25 | 0.155 | -0.59 | 0 | -0.55 | 0.04 |
| *EHD1* | 10938 | 1.64 | 0 | 1.05 | 0.333 |  |  |  |  |
| *FAM107B* | 83641 | 1.35 | 0.019 | 1.17 | 0.147 |  |  |  |  |
| *FAM60A* | 58516 | 1.32 | 0 | 1.23 | 0.25 |  |  |  |  |
| *FNDC3B* | 64778 | 1.73 | 0.003 | 1.21 | 0.049 |  |  |  |  |
| *FOSL2* | 2355 | 1.5 | 0.01 | 1.25 | 0.004 |  |  | -0.51 | 0.05 |
| *GBP3* | 2635 | 1.53 | 0.004 | 1.13 | 0.257 |  |  |  |  |
| *HBEGF* | 1839 | 1.61 | 0.022 | 1.03 | 0.443 |  |  |  |  |
| *ICAM1* | 3383 | 1.51 | 0.003 | 1.2 | 0.103 | -0.61 | 0 | -0.47 | 0.07 |
| *IFNGR1* | 3459 | 1.57 | 0.017 | 1.24 | 0.019 |  |  |  |  |
| *IRAK3* | 11213 | 1.72 | 0.011 | 1.24 | 3xE-04 |  |  |  |  |
| *KIF2A* | 3796 | 1.34 | 0.026 | 1.09 | 0.015 | -0.53 | 0.01 | -0.65 | 0.01 |
| *LAMB3* | 3914 | 1.84 | 0.046 | 1.28 | 0.006 | -0.55 | 0 |  |  |
| *LGR4* | 55366 | 1.4 | 0.035 | 1.16 | 0.253 |  |  |  |  |
| *LYN* | 4067 | 1.47 | 0.031 | 1.14 | 0.131 | -0.8 | 0 |  |  |
| *MAPK6* | 5597 | 1.45 | 0.025 | 1.08 | 0.444 |  |  |  |  |
| *MIER1* | 57708 | 1.31 | 0.017 | 1.01 | 0.929 |  |  |  |  |
| *MYO1B* | 4430 | 1.39 | 0.042 | 1.25 | 0.01 | -0.5 | 0.01 | -0.55 | 0.04 |
| *NAB1* | 4664 | 1.67 | 0 | 1.27 | 0.027 |  |  |  |  |
| *NAP1L1* | 4673 | 1.46 | 0.03 | 1.13 | 0.601 | -0.69 | 0 | -0.46 | 0.07 |
| *NCOA7* | 135112 | 1.7 | 0.01 | 1.2 | 0.038 |  |  |  |  |
| *NFKB1* | 4790 | 1.77 | 0.013 | 1.17 | 0.024 |  |  | -0.71 | 0.01 |
| *PALM2* | 445815 | 1.41 | 0.002 | 1.1 | 0.129 |  |  |  |  |
| *PIM2* | 11040 | 1.96 | 0.021 | 0.98 | 0.798 |  |  |  |  |
| *PRRG1* | 5638 | 1.45 | 0.039 | 1.22 | 0.132 |  |  |  |  |
| *RAC2* | 5880 | 1.34 | 0.008 | 1.2 | 0.017 | -0.75 | 0 |  |  |
| *RCAN1* | 1827 | 1.6 | 0.001 | 1.12 | 0.12 |  |  |  |  |
| *RNF24* | 11237 | 1.57 | 0.03 | 1.03 | 0.751 |  |  |  |  |
| *RPL15P17* | 728002 | 1.32 | 0.031 | 1.12 | 0.255 |  |  |  |  |
| *SAT1* | 6303 | 1.41 | 0.017 | 1.02 | 0.729 |  |  |  |  |
| *SDC4* | 6385 | 1.95 | 0 | 1.08 | 0.157 |  |  |  |  |
| *SERPINB8* | 5271 | 1.58 | 0.036 | 0.99 | 0.826 |  |  | -0.5 | 0.06 |
| *SERPINE1* | 5054 | 1.44 | 0.047 | 1.12 | 0.335 |  |  |  |  |
| *SGPP2* | 130367 | 1.68 | 0 | 1.18 | 0.05 |  |  |  |  |
| *SH3BP1* | 23616 | 1.43 | 0.001 | 1.14 | 0.068 |  |  |  |  |
| *SLC2A6* | 11182 | 1.42 | 0.009 | 1.05 | 0.269 |  |  |  |  |
| *SLC39A11* | 201266 | 1.45 | 0.005 | 1.2 | 0.083 |  |  |  |  |
| *SLC43A3* | 29015 | 1.36 | 0.007 | 1.11 | 0.131 | -0.56 | 0 |  |  |
| *SNX10* | 29887 | 1.34 | 0.001 | 1.06 | 0.184 |  |  |  |  |
| *SPATA5L1* | 79029 | 1.8 | 0.026 | 1.23 | 0.092 |  |  |  |  |
| *SRPK1* | 6732 | 1.42 | 0 | 1.1 | 0.213 |  |  |  |  |
| *STAT5A* | 6776 | 1.69 | 0.008 | 1.08 | 0.226 |  |  |  |  |
| *STOML1* | 9399 | 1.47 | 0.009 | 1.24 | 0.041 |  |  |  |  |
| *TDRD7* | 23424 | 1.33 | 0.004 | 1.12 | 0.161 |  |  |  |  |
| *TJP2* | 9414 | 1.34 | 0.004 | 1.16 | 0.13 |  |  |  |  |
| *TNFAIP3* | 7128 | 1.62 | 0.011 | 1.26 | 0.034 |  |  |  |  |
| *TRAM1* | 23471 | 1.32 | 0.017 | 1.1 | 0.113 | -0.5 | 0.01 | -0.49 | 0.06 |
| *TRIM16* | 10626 | 1.3 | 0.005 | 1.1 | 0.001 |  |  |  |  |
| *UBTD2* | 92181 | 1.45 | 0.004 | 1.18 | 0.013 |  |  |  |  |
| *ZC3H12A* | 80149 | 1.32 | 0.02 | 1.24 | 0.035 |  |  |  |  |
| *ZNF319* | 57567 | 1.38 | 0.049 | 1.26 | 0.043 |  |  |  |  |
| *ZNHIT6* | 54680 | 1.33 | 0.011 | 1.08 | 0.436 |  |  |  |  |
| *Genes up-regulated during 24hrs. exposure* | | | | | | |  |  |  |
| C3 | 718 | 1.27 | 0.075 | 1.6 | 0.011 | -0.78 | 0 | -0.6 | 0.02 |
| *CNIH* | 10175 | 1.27 | 0.009 | 1.4 | 0.012 |  |  |  |  |
| *CRYZ* | 1429 | 1.09 | 0.371 | 1.37 | 0.012 |  |  |  |  |
| *DHX15* | 1665 | 1.23 | 0.045 | 1.34 | 0.007 |  |  | -0.6 | 0.03 |
| *EBI3* | 10148 | 1.21 | 0.001 | 1.48 | 9xE-04 |  |  |  |  |
| *ELF3* | 1999 | 1.07 | 0.446 | 1.58 | 0.006 | -0.5 | 0.01 | -0.53 | 0.05 |
| *HSP90AA1* | 3320 | 1.23 | 0.069 | 1.4 | 0.003 |  |  |  |  |
| *LAMC2* | 3918 | 1.03 | 0.835 | 1.34 | 0.016 | -0.75 | 0 | -0.5 | 0.06 |
| *MMP1* | 4312 | 1.03 | 0.656 | 1.34 | 0.035 |  |  |  |  |
| *MMP7* | 4316 | 1.27 | 0.045 | 1.99 | 3xE-05 | -0.82 | 0 | -0.48 | 0.07 |
| *NFKBIA* | 4792 | 1.24 | 0.01 | 1.52 | 0.002 |  |  |  |  |
| *STAT1* | 6772 | 0.89 | 0.276 | 1.43 | 0.003 | -0.7 | 0 | -0.54 | 0.04 |
| *TMEM132A* | 54972 | 1.17 | 0.012 | 1.31 | 0.007 | -0.62 | 0 |  |  |
| *TNFSF10* | 8743 | 1.1 | 0.299 | 1.47 | 0.007 | -0.82 | 0 | -0.75 | 0 |
| *UGT2B7* | 7364 | 1.07 | 0.314 | 1.63 | 0.015 |  |  |  |  |
| *WFDC2* | 10406 | 1.12 | 0.197 | 1.31 | 0.019 | -0.79 | 0 | -0.65 | 0.02 |
| *Genes down-regulated during both exposures* | | | | | | |  |  |  |
| ABCC13 | 150000 | 0.52 | 0.006 | 0.6 | 0.057 |  |  |  |  |
| *AHR* | 196 | 0.58 | 0.006 | 0.49 | 0.026 |  |  |  |  |
| *AIRE* | 326 | 0.66 | 0.003 | 0.67 | 0.009 |  |  |  |  |
| *ANKLE1* | 126549 | 0.67 | 0.003 | 0.74 | 0.058 |  |  |  |  |
| *ANKRD44* | 91526 | 0.56 | 0.018 | 0.53 | 0.011 |  |  |  |  |
| *APOBEC3A* | 200315 | 0.62 | 0 | 0.61 | 0.037 |  |  |  |  |
| *ARSG* | 22901 | 0.61 | 0.003 | 0.64 | 0.074 |  |  |  |  |
| *ARSK* | 153642 | 0.6 | 0.003 | 0.61 | 0.003 |  |  |  |  |
| *ATG10* | 83734 | 0.66 | 0.003 | 0.68 | 0.039 |  |  |  |  |
| *BLZF1* | 8548 | 0.54 | 0.001 | 0.53 | 0.012 |  |  |  |  |
| *C2orf15* | 150590 | 0.63 | 0.005 | 0.63 | 0.003 |  |  |  |  |
| *C2orf56* | 55471 | 0.72 | 0.029 | 0.62 | 0.034 |  |  |  |  |
| *C4orf29* | 80167 | 0.62 | 0.023 | 0.59 | 0.046 |  |  |  |  |
| *C5orf24* | 134553 | 0.68 | 0.001 | 0.67 | 0.063 |  |  |  |  |
| *C5orf28* | 64417 | 0.53 | 0.003 | 0.58 | 0.03 |  |  |  |  |
| *C9orf80* | 58493 | 0.61 | 0.056 | 0.57 | 0.016 |  |  |  |  |
| *C9orf85* | 138241 | 0.65 | 0.001 | 0.68 | 0.063 |  |  |  |  |
| *CCDC30* | 728621 | 0.53 | 0 | 0.62 | 0.055 |  |  |  |  |
| *CEP135* | 9662 | 0.61 | 0 | 0.58 | 0.007 |  |  |  |  |
| *CEP19* | 84984 | 0.67 | 0.006 | 0.62 | 0.022 |  |  |  |  |
| *CHRNA5* | 1138 | 0.57 | 0.004 | 0.6 | 0.009 | 0.52 | 0.01 |  |  |
| *CREB1* | 1385 | 0.7 | 0.001 | 0.76 | 0.016 |  |  | 0.68 | 0.01 |
| *CSAD* | 51380 | 0.72 | 0.036 | 0.69 | 0.124 |  |  |  |  |
| *DBF4* | 10926 | 0.53 | 0.007 | 0.63 | 0.055 |  |  |  |  |
| *DIP2B* | 57609 | 0.5 | 0.003 | 0.54 | 0.015 |  |  |  |  |
| *DMC1* | 11144 | 0.59 | 0.022 | 0.64 | 0.125 | 0.57 | 0 |  |  |
| *DUSP19* | 142679 | 0.62 | 0.002 | 0.69 | 0.052 |  |  |  |  |
| *ERAP2* | 64167 | 0.61 | 0.003 | 0.67 | 0.05 |  |  |  |  |
| *EXOSC2* | 23404 | 0.69 | 0.007 | 0.63 | 0.021 |  |  |  |  |
| *FAM200A* | 221786 | 0.6 | 0.004 | 0.61 | 0.007 |  |  |  |  |
| *FAM217B* | 63939 | 0.7 | 0.04 | 0.63 | 0.002 |  |  |  |  |
| *FAM55B* | 120406 | 0.6 | 0.002 | 0.62 | 0.002 |  |  |  |  |
| *FAM73A* | 374986 | 0.57 | 0.006 | 0.61 | 0.033 |  |  |  |  |
| *FAM98B* | 283742 | 0.53 | 0.005 | 0.56 | 0.002 |  |  |  |  |
| *FBXL22* | 283807 | 0.55 | 0.008 | 0.55 | 0.011 |  |  |  |  |
| *FLJ25363* | 401082 | 0.63 | 0.003 | 0.58 | 0.047 |  |  |  |  |
| *FLJ39051* | 399972 | 0.65 | 0.006 | 0.53 | 0.002 |  |  |  |  |
| *FUT6* | 2528 | 0.67 | 0.002 | 0.74 | 0.002 | 0.65 | 0 |  |  |
| *FXYD2* | 486 | 0.68 | 0.049 | 0.7 | 0.032 |  |  |  |  |
| *GABPB2* | 126626 | 0.63 | 0.015 | 0.61 | 0.008 |  |  |  |  |
| *GNL3L* | 54552 | 0.7 | 0.033 | 0.66 | 0.014 |  |  |  |  |
| *GNPNAT1* | 64841 | 0.66 | 0.003 | 0.68 | 0.005 |  |  |  |  |
| *GOLGA2* | 2801 | 0.51 | 0.012 | 0.56 | 0.008 |  |  | 0.67 | 0.01 |
| *GPR1* | 2825 | 0.6 | 0.007 | 0.53 | 0.022 |  |  |  |  |
| *GPR155* | 151556 | 0.75 | 0.034 | 0.66 | 0.021 |  |  |  |  |
| *HEATR5A* | 25938 | 0.57 | 0.001 | 0.56 | 0.026 |  |  |  |  |
| *HIPK2* | 28996 | 0.7 | 0 | 0.71 | 0.023 | 0.51 | 0.01 |  |  |
| *HSD17B7* | 51478 | 0.56 | 0.006 | 0.63 | 0.026 |  |  |  |  |
| *HSPB1P2* | 653364 | 0.7 | 0.035 | 0.76 | 0.027 |  |  |  |  |
| *ID2* | 3398 | 0.67 | 0.05 | 0.69 | 0.004 |  |  |  |  |
| *IFT88* | 8100 | 0.63 | 0.023 | 0.58 | 0.003 |  |  |  |  |
| *ITCH* | 83737 | 0.67 | 0.006 | 0.72 | 0.012 |  |  |  |  |
| *KBTBD6* | 89890 | 0.59 | 0.01 | 0.63 | 0.035 |  |  |  |  |
| *KCNH6* | 81033 | 0.59 | 0.006 | 0.56 | 0.01 | 0.45 | 0.02 | 0.74 | 0 |
| *KLHL28* | 54813 | 0.58 | 0.001 | 0.55 | 0.083 |  |  |  |  |
| *LOC285696* | 285696 | 0.5 | 0.002 | 0.49 | 0.076 |  |  |  |  |
| *LOC286177* | 286177 | 0.64 | 0.003 | 0.6 | 0.06 |  |  |  |  |
| *LOC51145* | 51145 | 0.62 | 0.011 | 0.58 | 0.031 |  |  |  |  |
| *LRRC2* | 79442 | 0.61 | 0.012 | 0.64 | 0.003 |  |  |  |  |
| *LRRFIP1* | 9208 | 0.71 | 0.03 | 0.75 | 0.053 |  |  |  |  |
| *MCM8* | 84515 | 0.64 | 0.029 | 0.63 | 0.022 |  |  |  |  |
| *METTL21A* | 151194 | 0.59 | 0.002 | 0.66 | 0.035 |  |  |  |  |
| *MGC13053* | 84796 | 0.55 | 0.004 | 0.53 | 0.027 |  |  |  |  |
| *MGC16703* | 113691 | 0.51 | 0.004 | 0.56 | 0.018 |  |  |  |  |
| *MICAL3* | 57553 | 0.58 | 0 | 0.55 | 0.011 |  |  |  |  |
| *MRP63* | 78988 | 0.66 | 0.017 | 0.63 | 0.049 | 0.51 | 0.01 |  |  |
| *MRTO4* | 51154 | 0.71 | 0.004 | 0.77 | 0.035 |  |  |  |  |
| *MSH3* | 4437 | 0.6 | 0.006 | 0.61 | 0.033 |  |  | 0.63 | 0.02 |
| *MSRB3* | 253827 | 0.65 | 0.02 | 0.65 | 0.019 |  |  |  |  |
| *N4BP2* | 55728 | 0.65 | 0.003 | 0.63 | 0.017 |  |  |  |  |
| *NARF* | 26502 | 0.58 | 0.018 | 0.62 | 0.036 |  |  |  |  |
| *ND1* | 4535 | 0.75 | 0.174 | 0.75 | 0.013 |  |  |  |  |
| *NDUFC2* | 4718 | 0.72 | 0.014 | 0.7 | 0.058 |  |  |  |  |
| *NMNAT1* | 64802 | 0.66 | 0.011 | 0.66 | 0.037 |  |  |  |  |
| *NPIPL3* | 23117 | 0.73 | 0.055 | 0.59 | 0.039 |  |  |  |  |
| *NT5C2* | 22978 | 0.68 | 0.004 | 0.64 | 0.017 |  |  |  |  |
| *NUBPL* | 80224 | 0.59 | 0.002 | 0.61 | 0.042 |  |  |  |  |
| *NWD1* | 284434 | 0.62 | 0.002 | 0.56 | 0.016 |  |  |  |  |
| *OLAH* | 55301 | 0.6 | 0.003 | 0.57 | 0.017 |  |  |  |  |
| *OVOL2* | 58495 | 0.6 | 0.009 | 0.53 | 0.02 |  |  |  |  |
| *PBOV1* | 59351 | 0.67 | 0.032 | 0.71 | 0.086 | 0.55 | 0 |  |  |
| *PDLIM5* | 10611 | 0.64 | 0.003 | 0.64 | 0.024 |  |  | 0.53 | 0.05 |
| *PNPLA8* | 50640 | 0.63 | 0.017 | 0.56 | 0.042 |  |  |  |  |
| *PXN* | 5829 | 0.72 | 0.027 | 0.64 | 0.015 |  |  |  |  |
| *RAD51B* | 5890 | 0.61 | 0.009 | 0.59 | 0.004 |  |  |  |  |
| *RGPD4* | 285190 | 0.7 | 0.007 | 0.7 | 0.011 |  |  |  |  |
| *RPL4P5* | 158345 | 0.65 | 0.033 | 0.67 | 0.065 |  |  |  |  |
| *RPSA* | 3921 | 0.76 | 0.026 | 0.76 | 0.187 |  |  |  |  |
| *RSU1* | 6251 | 0.59 | 0.02 | 0.63 | 0.023 |  |  |  |  |
| *SCIN* | 85477 | 0.61 | 0.056 | 0.59 | 0.005 |  |  |  |  |
| *SCN11A* | 11280 | 0.71 | 0.002 | 0.72 | 0.053 |  |  |  |  |
| *SHCBP1* | 79801 | 0.65 | 0.002 | 0.6 | 0.011 |  |  |  |  |
| *SLC26A4* | 5172 | 0.54 | 0.001 | 0.52 | 0.018 | 0.58 | 0 |  |  |
| *SLC44A4* | 80736 | 0.67 | 0.022 | 0.64 | 0.015 |  |  |  |  |
| *SLC7A11* | 23657 | 0.63 | 0.021 | 0.62 | 0.023 |  |  |  |  |
| *SLFN13* | 146857 | 0.58 | 0.003 | 0.54 | 0.027 |  |  |  |  |
| *SMG1* | 23049 | 0.62 | 0.005 | 0.6 | 0.023 |  |  | 0.53 | 0.05 |
| *SMN1* | 6606 | 0.71 | 0.057 | 0.72 | 0.04 |  |  |  |  |
| *SRRM2* | 23524 | 0.77 | 0.006 | 0.76 | 0.014 |  |  |  |  |
| *STXBP4* | 252983 | 0.7 | 0.036 | 0.74 | 0.032 |  |  |  |  |
| *SUV39H2* | 79723 | 0.59 | 0.001 | 0.5 | 0.001 |  |  |  |  |
| *SWSAP1* | 126074 | 0.56 | 0.003 | 0.55 | 0.015 |  |  |  |  |
| *SYAP1* | 94056 | 0.62 | 0.006 | 0.56 | 0.013 |  |  |  |  |
| *SYDE2* | 84144 | 0.63 | 0.01 | 0.7 | 0.166 |  |  |  |  |
| *TADA3* | 10474 | 0.58 | 0.009 | 0.6 | 0.044 |  |  |  |  |
| *TEP1* | 7011 | 0.69 | 0.009 | 0.71 | 0.092 |  |  |  |  |
| *TMBIM4* | 51643 | 0.7 | 0.008 | 0.63 | 0.018 |  |  |  |  |
| *TMEM17* | 200728 | 0.63 | 0.005 | 0.71 | 0.012 |  |  |  |  |
| *TMSB4X* | 7114 | 0.58 | 0.015 | 0.72 | 0.138 |  |  |  |  |
| *TMSB4XP1* | 7115 | 0.63 | 0.013 | 0.74 | 0.055 |  |  |  |  |
| *TMSB4XP2* | 7116 | 0.58 | 0.005 | 0.71 | 0.123 |  |  |  |  |
| *TMSB4XP6* | 7120 | 0.56 | 0.01 | 0.7 | 0.107 |  |  |  |  |
| *TNFRSF6B* | 8771 | 0.76 | 0.116 | 0.76 | 0.038 |  |  |  |  |
| *TRIM58* | 25893 | 0.6 | 0.011 | 0.65 | 0.004 |  |  |  |  |
| *TRIM74* | 378108 | 0.5 | 0.008 | 0.54 | 0.029 |  |  |  |  |
| *TTF2* | 8458 | 0.65 | 0.01 | 0.65 | 0 |  |  |  |  |
| *TUBGCP4* | 27229 | 0.62 | 0.024 | 0.59 | 0.079 |  |  |  |  |
| *VSIG1* | 340547 | 0.68 | 0.03 | 0.58 | 0.031 |  |  |  |  |
| *XIAP* | 331 | 0.66 | 0.021 | 0.64 | 0.058 |  |  |  |  |
| *XPNPEP3* | 63929 | 0.59 | 0.014 | 0.55 | 0.043 |  |  |  |  |
| *ZMAT3* | 64393 | 0.63 | 0.021 | 0.66 | 0.01 |  |  |  |  |
| *ZNF14* | 7561 | 0.53 | 0.007 | 0.53 | 0.017 |  |  |  |  |
| *ZNF33A* | 7581 | 0.62 | 0.011 | 0.55 | 0.039 |  |  |  |  |
| *ZNF417* | 147687 | 0.51 | 0.006 | 0.48 | 0.003 |  |  |  |  |
| *ZNF430* | 80264 | 0.67 | 0 | 0.72 | 0.022 |  |  |  |  |
| *ZNF454* | 285676 | 0.67 | 0.039 | 0.64 | 0.034 |  |  |  |  |
| *ZNF542* | 147947 | 0.61 | 0.003 | 0.57 | 0.022 |  |  |  |  |
| *ZNF549* | 256051 | 0.58 | 0.002 | 0.6 | 0.007 |  |  |  |  |
| *ZNF620* | 253639 | 0.71 | 0.009 | 0.75 | 0.12 |  |  |  |  |
| *ZNF623* | 9831 | 0.63 | 0.013 | 0.6 | 0.026 |  |  |  |  |
| *ZNF623* | 9831 | 0.57 | 0.003 | 0.53 | 0.027 |  |  |  |  |
| *ZNF652* | 22834 | 0.65 | 0.016 | 0.59 | 0.04 |  |  |  |  |
| *ZNF665* | 79788 | 0.64 | 0.003 | 0.72 | 0.017 |  |  |  |  |
| *ZNF667* | 63934 | 0.7 | 0.017 | 0.74 | 0.054 |  |  |  |  |
| *ZNF786* | 136051 | 0.59 | 0.006 | 0.61 | 0.077 |  |  |  |  |
| *ZNF791* | 163049 | 0.74 | 0.047 | 0.71 | 0.005 |  |  |  |  |
| *ZNF91* | 7644 | 0.68 | 0.026 | 0.61 | 0.045 |  |  |  |  |
| *ZNF98* | 148198 | 0.66 | 0.005 | 0.61 | 0.064 |  |  |  |  |
|  | *Genes down-regulated during 6hrs. exposure* | | | | |  |  |  |  |
| ABCA7 | 10347 | 0.72 | 0.031 | 0.93 | 0.315 |  |  |  |  |
| *ATP6* | 4508 | 0.72 | 0.043 | 0.87 | 0.251 |  |  |  |  |
| *C4orf34* | 201895 | 0.68 | 0.01 | 0.79 | 0.005 |  |  |  |  |
| *CABLES1* | 91768 | 0.77 | 0 | 1.01 | 0.767 |  |  |  |  |
| *CASP10* | 843 | 0.74 | 0.014 | 1.05 | 0.317 |  |  |  |  |
| *CASP6* | 839 | 0.75 | 0.032 | 0.78 | 0.141 |  |  |  |  |
| *CATSPER2* | 117155 | 0.65 | 0.001 | 0.78 | 0.079 |  |  |  |  |
| *CDC14B* | 8555 | 0.73 | 0.007 | 0.99 | 0.744 |  |  | 0.77 | 0 |
| *CDC25B* | 994 | 0.74 | 0.007 | 0.86 | 0.118 |  |  |  |  |
| *CTDSPL* | 10217 | 0.62 | 0.001 | 0.92 | 0.581 |  |  |  |  |
| *CXXC5* | 51523 | 0.76 | 0.006 | 0.88 | 0.11 |  |  |  |  |
| *DHRS3* | 9249 | 0.75 | 0.015 | 0.94 | 0.341 | 0.5 | 0.01 |  |  |
| *FOXL2* | 668 | 0.74 | 0.002 | 0.86 | 0.164 |  |  |  |  |
| *GGCT* | 79017 | 0.76 | 0.012 | 0.93 | 0.62 |  |  |  |  |
| *GPRC5C* | 55890 | 0.7 | 0.003 | 1.02 | 0.562 |  |  |  |  |
| *GTF3C6* | 112495 | 0.75 | 0.003 | 0.97 | 0.372 |  |  |  |  |
| *HAUS2* | 55142 | 0.62 | 0.002 | 0.78 | 0.103 |  |  |  |  |
| *IER5L* | 389792 | 0.75 | 0.003 | 0.84 | 0.075 |  |  |  |  |
| *INTS3* | 65123 | 0.76 | 0.027 | 0.86 | 0.102 |  |  |  |  |
| *KANK2* | 25959 | 0.71 | 0 | 0.86 | 0.009 |  |  |  |  |
| *MAP3K9* | 4293 | 0.77 | 0.042 | 0.85 | 0.059 | 0.53 | 0.01 |  |  |
| *MITF* | 4286 | 0.74 | 0.03 | 0.94 | 0.414 |  |  |  |  |
| *NAT14* | 57106 | 0.71 | 0.016 | 0.97 | 0.497 |  |  |  |  |
| *ND2* | 4536 | 0.7 | 0.017 | 0.81 | 0.047 |  |  |  |  |
| *NFAT5* | 10725 | 0.69 | 0.006 | 0.93 | 0.584 |  |  |  |  |
| *NFATC3* | 4775 | 0.56 | 0.003 | 0.95 | 0.498 |  |  | 0.66 | 0.01 |
| *NPR1* | 4881 | 0.72 | 0.027 | 0.81 | 0.005 |  |  |  |  |
| *ODZ3* | 55714 | 0.76 | 0.019 | 0.93 | 0.338 |  |  |  |  |
| *PCSK9* | 255738 | 0.75 | 0.008 | 0.88 | 0.176 |  |  |  |  |
| *PIP4K2B* | 8396 | 0.65 | 0.008 | 0.99 | 0.88 |  |  | 0.6 | 0.02 |
| *PPME1* | 51400 | 0.77 | 0.012 | 0.94 | 0.117 |  |  |  |  |
| *RPL21* | 6144 | 0.75 | 0.031 | 0.86 | 0.108 |  |  |  |  |
| *RPL5P7* | 344178 | 0.7 | 0.047 | 0.8 | 0.165 |  |  |  |  |
| *S100A4* | 6275 | 0.76 | 0.013 | 0.86 | 0.288 |  |  |  |  |
| *SLC2A3* | 6515 | 0.74 | 0.007 | 0.78 | 0.012 | 0.52 | 0.01 |  |  |
| *SLC48A1* | 55652 | 0.77 | 0.001 | 0.81 | 0.026 |  |  |  |  |
| *SMAP1* | 60682 | 0.72 | 0.007 | 1.12 | 0.114 |  |  |  |  |
| *TTLL12* | 23170 | 0.73 | 0.003 | 0.98 | 0.655 |  |  |  |  |
| *TUBA3C* | 7278 | 0.72 | 0.009 | 0.85 | 0.381 |  |  |  |  |
| *UQCRH* | 7388 | 0.74 | 0.008 | 0.87 | 0.146 |  |  |  |  |
| *USP14* | 9097 | 0.76 | 0.013 | 0.78 | 0.069 |  |  |  |  |
| *VASN* | 114990 | 0.66 | 0.009 | 1.05 | 0.081 |  |  |  |  |
| *WDR82* | 80335 | 0.74 | 0.006 | 0.8 | 0.022 |  |  |  |  |
| *YIPF2* | 78992 | 0.77 | 0.006 | 0.83 | 0.11 |  |  |  |  |
| *ZNF608* | 57507 | 0.71 | 0.004 | 1.05 | 0.444 |  |  |  |  |
| *Genes down-regulated during 24hrs. exposure* | | | | | |  |  |  |  |
| COX2 | 4513 | 0.85 | 0.238 | 0.67 | 0.011 |  |  |  |  |
| *DLC1* | 10395 | 1.29 | 0.284 | 0.6 | 0.005 |  |  | 0.55 | 0.04 |
| *DUSP1* | 1843 | 1.02 | 0.608 | 0.76 | 0.044 | 0.44 | 0.02 |  |  |
| *DUSP5* | 1847 | 0.98 | 0.436 | 0.73 | 0.01 |  |  |  |  |
| *ID1* | 3397 | 0.9 | 0.09 | 0.62 | 0.001 |  |  |  |  |
| *ITGB3* | 3690 | 0.93 | 0.566 | 0.77 | 0.018 | 0.58 | 0 |  |  |
| *MACF1* | 23499 | 0.91 | 0.164 | 0.74 | 0.028 |  |  |  |  |
| *MT1JP* | 4498 | 0.89 | 0.106 | 0.68 | 0.037 |  |  |  |  |
| *PRSS23* | 11098 | 0.84 | 0.036 | 0.76 | 0.002 |  |  |  |  |
| *STRA6* | 64220 | 0.99 | 0.193 | 0.74 | 0.002 | 0.69 | 0 |  |  |
| *TNFRSF12A* | 51330 | 0.88 | 0.066 | 0.76 | 0.006 |  |  |  |  |
| *TNFRSF6B* | 8771 | 0.86 | 0.095 | 0.76 | 0.008 |  |  |  |  |
